# Sick Headache Cured by Full Inspirations

**Published:** 1851-03

**Authors:** 


*Sick Headache cured by Full Inspirations.*—When a medication is based upon the experiments made upon himself by an honorable professional brother, it is far better, in reporting it, to give his own words. We will then simply publish the communication of M. Tavignot upon the new therapeutic agent in the cure of this painful, if not dangerous disease.

"It was in the following manner that I discovered the efficacy of this new and apparently strange method for the cure of this affection. In October last I was attacked with pain and weight in the head, anorexia, a physical and moral prostration, etc. Experience taught me that I had to remain in this state for twenty-four hours. I concluded that this peculiar state of the nervous centers might depend upon a stagnation of blood in the venous sinuses of the dura-mater, as M. Auzias Turenne supposes, or upon an imperfect æration of this fluid. I immediately commenced respiring freely and fully during several minutes. I perceived a sensible relief, which induced me to continue, and in a short time I was cured. I got up and undertook my usual occupations, as I felt but a slight pain in my temples, which vanished in a quarter of an hour. This result was doubly agreeable to me, as it furnished me with a new and practical remedy. In ten persons, upon whom it has been tried, one-half have found an instantaneous relief, and in the others there has been an amelioration, or a complete failure. However, upon interrogating with care those who were not relieved, I am convinced that they did not have genuine sick headache: they had a neuralgic pain of the head, but it was not accompanied with that profound prostration and melancholy that I have mentioned as characteristic of the disease. It seems to me to be useless to search for the modus operandi of full and profound inspirations in the cure of sick headache. It is evident that by this means the venous circulation is accelerated, and the chemico-physiological act of hematosis is hastened. Then the explanation of the success of this new method is in one or the other of these conditions, or perhaps in both.—*L'Observation*, from *Southern Med. and Surg. Jour.*

